# 

**Published:** 1901-12

**Authors:** 


### PERSONALS.

Dr. M. M. White, of Monroe, La., has entered the Chicago Veterinary College.

Dr. A. F. Schrieber was a visitor to Western Pennsylvania in October, on official business of the State Live-stock Sanitary Board.

Dr. J. O. George, of Camden, N. J., will erect a commodious veterinary hospital in connection with his veterinary practice.

Dr. A. S. Wheeler, of Baltimore Farms, Asheville, N. C., was a visitor to Philadelphia in November. He was entertained at dinner by Dr. John W. Adams, who also had as a guest Dr. Leonard Pearson.

Dr. C. H. Magill, of Philadelphia, Pa., though no longer in the active pursuit of his profession, still loves a good horse, and now and again holds the ribbons over a fast one at our local driving tracks when friends are trying the mettle of their favorite steeds.

Dr. James T. McAnulty, of Philadelphia, continues to take an active part in all matters of interest to the horseshoers.

Dr. Joseph S. Cattanach, of New York, has an interesting letter of his travels abroad in the November 9, 1901, issue of the *Rider and Driver.*

Dr. J. A. Atkinson, of Goodwill Hill, Pa , is taking a trip South for a month or so.

Dr. W. B. Craig, of Indianapolis, Ind., tells an interesting story of how he sewed up a long, incised wound on an ostrich's neck, received in transit from the East to his Western ranch home.

Dr. H. H. Weinberg, of Philadelphia, with his trotter "Silver Heels," won the 2.27 race at Belmont Park, October 31st. Dr. Weinberg drives his own speedy ones, and in this race was to a road-cart.

Dr. C. M. McFarland, of the Bureau of Animal Industry, recently located at Cincinnati, Ohio, is now at Billings, Mont.

Dr. A. J. Sheldon, of Boston, Mass., in relinquishing his connection with the Harvard Veterinary Department, where he was in active pursuit of veterinary medicine, will associate himself with one of New York's financial institutions in the future.

### INDEX

ABDOMINAL section, 477
Abscess, polynuclear hyperleucocytosis in diagnosing liver, 724
Abscesses and fistulæ in arthritis of the foot, seat of, 576
Absorption of various drugs by the bladder, 719
Acid for detecting sugar in urine, orthonitrophenylpropionic, 571
Abstract from Professor Hunting's remarks on pelvic fractures in the horse, 744
Ackerman, E. B., 647
Actinomycosis, iodide of potassium in treatment of human, 567
in man, 636
of the bladder, primary, 384.
Action of solar and diffuse lights upon the tubercle bacillus in sputum, the, 572
Actions and uses of many of the alkaloids, uncertainty surrounding the, 167
Actual cautery in treatment of cartilaginous quittor, 148
Acute polyarthritis in the cow, 384
Address at the Iowa and Nebraska Veterinary Medical Association, President's, 368
the President's, 635, 713
Adeno-tuberculosis, shoulder lameness in cattle from subscapular, 570
Adulteration, drug-, 94
of cinnamon bark, 718
Affection in the dog and the cat, a gastro-intestinal, 575
Alcohol as a disinfectant, 241
Alkalies in rheumatic fever, the salicylates and, 306
Alkaloids, formation of cinchona, 424
uncertainty surrounding the actions and uses of many of the, 167
Allegheny County Veterinary Medical Association, 673, 743
Alumni Association of the Veterinary Department of the New York University, 202, 337
Veterinary Department University of Pennsylvania, Society of the, 539
America, National Horse Show Association of, 650
American Veterinary Medical Association, 737
37th Annual Convention, Detroit, 55
38th Annual Convention, Atlantic City, 250, 377, 386, 437, 441, 450, 508, 528, 569, 655
Committee on Diseases, 800
Treasurer's report of the, 810
Among the Colleges, 255, 431
Legislators, 175
Amyl salicylate, 305
Anæsthesia of the spinal cord, cocaine, 725
Anæsthetic, chloretone as a local, 723
in the horse, chloral as an, 308
Aneurism of the inferior cervical artery, 515
Animals, diseases common to man and, 24
experimental tuberculosis, human and bovine, in the domestic, 33
Annales de Med. Vet., 308, 384, 385, 432, 433, 570, 572, 577, 639, 792
Anthrax and preventive inoculation in Louisiana, 613, 708
Antidote for morphine poisoning, a new, 563
Antipyrine, elimination of, 567
Antisepsis in strangles infection, pulmonary, 435
Antiseptic soaps, 514
solution, Burrow's, 95
Antiseptics, a comparison of, 720
Antitoxin, preservatives in, 242
Aponeuroses of the thigh, osteoma of the, 511
Aphthous fever to the horse, transmission of, 384
Apoplexy, iodide of potassium in parturient, 432
Application of light, therapeutic, 434
Artery, aneurism of the inferior cervical, 515
Arthritis of the foot, seat of abscesses and fistulæ in, 576
pneumococcic, 243
protargol in purulent, 513
tubercular, 169
Army, equine diseases in the Prussian, 788
improved Veterinary service, United States, 57, 114, 190, 252
Veterinary Department, 804
Arteries, suture of, 169
Arterio-sclerosis in the spinal cord, 790
Artery, a case of thrombosis of the pulmonary, 797
Articular rheumatism, phenol in acute, 564
Ass contract tuberculosis? can the, 308
Association Meetings, 473, 534, 598, 674
Atlantic City, 1901, 587, 648
Atrophy of the pancreas, diabetic urine from complete, 512
semimembranous muscle following castration, 512
Attitude of some shorthorn breeders toward the tuberculin test, and the animus thereof, the, 109
Autopsy, report of an, 597
Azoturia, treatment of, 170

BACILLI by cows in coughing, a possible source of contagion, the dissemination of tubercle, 15
Bacillus from infectious vulvar disease of cattle, a, 92
in sputum, the action of solar and diffuse light upon the tubercle, 572
Bacteria, dairy, 427
to heat and cold, resistance of, 241
Balsam Peru, production of, 785
Banquet, the JOURNAL, 315
Bark, adulteration of cinnamon, 718

Beef measles, 132
Benign tumors and cysts, 361
Better times now, the, 440
Bicipital synovitis, 570
Bilateral cryptorchism, a case of, 423
Bitch after oöphorectomy, on the recurrence of estruation in the, 284
extra-uterine fœtation in the, 726
Bladder, absorption of various drugs by the, 719
primary actinomycosis of the, 384
Board of Veterinary Medical Examiners, 599
Boards, our State, 794
Boiled milk, testing crude and, 724
Boer war, horses in the, 464
Book review, self-examination, 303
Bovidæ, transmission of sarcoptes minor from the cat to, 574
Bowels and heat stroke, catarrh of the, 517
Breeding, the principles of stock, 226
Breeders toward the tuberculin test and the animus thereof, the attitude of some shorthorn, 109
British congress on tuberculosis, the, 586
Brood mares, the management of, 158
Brown, J. E., M.D., 368
Bruises of the pododerm, 40
Budd, T. Earle, 495
Bullock, post-mortem examination of a cryptorchid, 389
Bull. Pharmacy, 244, 563, 718
Bureau of Animal Industry, Promotions of Veterinarians, 587
Burden, Charles, resolutions on the death of, 662
Burns, hydrogen dioxide for, 789
Burrow's antiseptic solution, 95
Bursal enlargements, radical operations for, 691
Butler, Tait, 632, 713

Cæsarean section on a cow, 444
Calves, etiology of infectious enteritis and bronchopneumonia in, 574
Campbell, W. T., V. S., 150
Canine distemper, 638
experimental vaccination against, 572
instruments, some new, 161
Care and treatment of the horse's foot, a few suggestions as to, 295
Carbon detergen, liquor, 718
Carter, J. M., V.M.D., 696
Cartilaginous quittor, actual cautery in the treatment of, 148
Cases of hock lameness, neurectomy in, 430
Castration, atrophy of the semimembranous muscle following, 512
persistence of the genesic instinct after incomplete, 307
tumors in swine, 233
Cat, gastro-intestinal affection in the dog and the, 575
the use of thermol in influenza and distemper in the dog and, 556
to bovidæ, transmission of sarcoptes minor from the, 574
Catarrh of the bowels and heat stroke, 517
Cattle, a bacillus from infectious vulvular disease of, 92
in India, tuberculosis among, 168
infectious ulcer of the vulva of, 89
inspection and the tuberculin test, 144
transportation, regulations concerning, 739
tuberculosis of man and, 521
Cause and treatment of periodic ophthalmia, 573
Celiotomy, laparotomy, 477
Cervical artery, aneurism of the inferior, 515
Chicago Veterinary College, 258, 266, 431
Chickens, destruction of vermin on, 513
Chipman, W. W., M.D., 726
Chirol, 571
Chloral as an anæsthetic in the horse, 308
Chloretone as a local anæsthetic, 723
Cinchona alkaloids, formation of, 424
Cinnamon bark, adulteration of, 718
Cities, foot-sore horses in our, 415
Clark, W. G., 537, 598
Clement, A. W., necrology, 196
resolutions on the death of, 662
Clinical reports, 170
study of the symptoms of shoulder lameness, 381
Cocaine anæsthesia of the spinal cord, 725
Coffee, P. D., resolutions on the death of, 662
Cold, resistance of bacteria to heat and, 241
Colleges, among the, 255, 431
and Associations as guards of our standing among the profession, 759
veterinary organizations and ill-equipped veterinary, 112
Colon into the stomach, transplantation of a piece of, 429
Commencement exercises—
Chicago Veterinary College, 266
Grand Rapids Veterinary College, 263
Kansas City Veterinary College, 265
McGill University, Veterinary Department, 332
McKillip Veterinary College, 263
New York American Veterinary College, 464
Ontario Veterinary College, 54, 258
University of Pennsylvania, Veterinary Department, 533
Western Veterinary College, 265
Commission, a royal, 786
Committee on Army Legislation, report of, 746
on Diseases, A. V. M. A., 800
on Intelligence and Education, report of, 488
reports, A. V. M. A., 664
Communications, 319, 392, 450, 527, 592, 649, 739
Compound comminuted fracture with indirect simple fracture of the inferior maxilla direct, 44
Concerning inguinal hernia in the female dog, 409
Congress of Medicine, first Egyptian, 654
on tuberculosis, the British, 586
Control work, 591
Constituents, digitalis and its active, 502
Copeman, S. Monckton, M.A., M.D., F.R.C.P., 802
Cord, arterio-sclerosis in the spinal, 790
Cork, loosen the, 240
Corns, 40
Corrections, 54, 674
Correspondence, 48, 100, 172, 246, 324, 394, 452, 528, 594, 651, 800
Cow, acute polyarthritis in the, 384
Cæsarean section on a, 444
in coughing, a possible source of contagion, the dissemination of tubercle bacillus by, 15
in reducing eversion of the uterus, position of the, 576
pulmonary emphysema in a, 47
which have suffered from mammitis, persistence of microbes in milk of, 430
Crowforth, Anderson, 337, 601
Crude and boiled milk, testing, 724
Cryptorchid bullock, post-mortem examination of a, 389
Cryptorchism, a case of bilateral, 423
Cynanche parotidæ, 383
Cysts, benign tumors and, 361
Cyst on the omentum, dermoid, 360

Dairy bacteria, 427
Dalrymple, W. H., M.R.C.V.S., 614, 664, 708, 740

Dawson, Charles F., M.D., D.V.S., 731
Death of Dr. Huidekoper, Rose Tree resolutions on the, 796
of George Fleming, the, 310
Defeat such legislation, 176
Defence of the Ontario Veterinary College, 172
Department of canine, feline, and avian medicine and surgery—
Bilateral cryptorchism, a case of, 423
Canine instruments, some new, 161
Dentition of two Mexican hairless dogs, 500
Experimental rectal injection in the dog, 373
Formation of cinchona alkaloids, 424
Fracture with indirect simple fracture of the inferior maxilla, direct compound, 44
Multiple pure lipomata in a parrot, 299
Puerperal septic metritis, 498
Rinderpest in the Philippines, 300
Systemic secretion of the testicle, 501
Uterine wall and purulent peritonitis, perforation of, 498
Department of surgery—
Hoof diseases, 40, 165, 238, 301, 375, 425
Dermoid cyst of the omentum, 360
Desmond, veterinary surgeon, 69
Destruction of vermin on chickens, 513
Detroit, September, 1900, Thirty-seventh Annual Convention, A.V.M.A., 55
Diabetic urine from complete atrophy of the pancreas, 512
Diaphragm, rupture of, 391
Digestive tract, tetanic infection through the, 717
Digitalis and its active constituents, 502
Dinwiddie, R. R., M.D., V.S., 33, 801
Diphtheria in the horse, 360
Direct compound comminuted fracture with indirect simple fracture of the inferior maxilla, 44
Disease of cattle, a bacillus from infectious vulvar, 92
Diseases common to man and animals, 24
hoof, 40, 165, 238, 375, 425
Disgrace, a national, 518
Disinfectant, alcohol as a, 241
Disorders of the horse, gastric and intestinal, 69
Dissemination of tubercle bacilli by cows in coughing, a possible source of contagion, 15
Distemper, canine, 638
in the dog, 703
and cat, the use of thermol in influenza and, 556
treatment of, 790
Doe, John, 597
Dog and cat, gastro-intestinal affection in the, 575
the use of thermol in influenza and distemper in the, 556
and the production of distemper vaccine, 802
concerning inguinal hernia in the female, 409
distemper in the, 703
experimental rectal injection in the, 373
mange or scabies in the, 150
mumps in the, 383
the micro-organism of distemper in the, 802
the thyroid gland and thyroid glandules of the, 1
unusual number of tapeworms in a, 788
Dogs in health, on the feeding of, 137
the dentition of two Mexican hairless, 500
Domestic animals, experimental tuberculosis, human and bovine, in the, 33
Double tibic peroneal neurectomy, 298
Dougherty, W. H., 665
Drasky, J. J., 335
Drug adulteration, 94
Drury, J., V.S., 560
Duncan, H., M.D., 233
Dystokia, 420
maternal, 495
Editorials, 113, 587, 737, 795
A.V.M.A., 737
Annual meeting of, 386, 441
Army veterinary service, 252
Atlantic City, 1901, 587
Be up and doing, 53
Better times now, the, 440
Closing of the year, the, 791
Defeat such legislation, 176
Fleming, George, death of, 310
Huidekoper, Rush Shippen, 793
Illinois, Dr. Swain's letter, 310
Just what should have been done, 388
Journal, how we would run a, 736
Journal's welcome, the, 524
Legislation, defeat such, 176
Legislative harvest, the, 387
McKinley, William, 645
Montana, well done! well done, Knowles, 176
National disgrace, a, 518
Our 1901 meeting, 648
Pennsylvania, well done, 310
Promotions of veterinarians in the bureau of animal industry from December 1, 1900, to July 31, 1901, 587
Recognition, a well merited, 254
Short-horn breeders toward the tuberculin test, and the animus thereof, the attitude of, 109
Senior editor of the Journal to Philadelphia, return of the, 309
State boards, our, 794
Tuberculosis of man and cattle, 521
the British congress on, 586
Veterinary register, 438
organizations move in the matter, let the, 112
Wasted sympathy, 646
Where in 1902, 794
Year, the closing of the, 793
Education in Spain, professional, 590
Effects of mammary injections of iodide of potash, 532
of salicylates upon metabolism, 567
Eger, Alex., 53
Egyptian congress of medicine, first, 654
Eichhorn, Dr., 202, 338
Elimination of antipyrine, 567
Emphysema in a cow, pulmonary, 47
Enlargement, radical operations for, 691
Enteritis and bronchopneumonia in calves, etiology of infectious, 574
Ephemeris, the, 304
Epicarin in the treatment of scabies, 78
Epizootic ophthalmia in the ox, etiology of, 385
Equine diseases in the Prussian army, 788
Ergotism, forage-poisoning, 446
Essence of turpentine, treatment of lameness by the subcutaneous injection of, 379
Estruation in the bitch after oöphorectomy, on the recurrence of, 284
Etiology and treatment of periodic ophthalmia, 433
spavin, 80
yellow fever, 305
Extra-uterine fœtation in the bitch, 726
Eversion of the uterus, position of the cow in reducing, 576
Examination for meat inspector, 53
Examinations, Michigan State Board, 393
Pennsylvania State Board of Veterinary Medical, 393
Experimental rectal injections in the dog, 373
tuberculosis, human and bovine, in the domestic animals, 33
vaccination against canine distemper, 572

Explosion, disastrous, 240
Extracts from foreign journals (French)—
Action of solar and diffuse light upon the tubercle bacillus in sputum, 572
Acute polyarthritis in the cow, 384
Aneurism of the inferior cervical artery, 515
Antiseptic soaps, 514
Arterio-sclerosis in the spinal cord, 788
Aphthous fever to the horse, transmission of, 384
Ass contract tuberculosis, can the, 308
Atrophy of the semimembranous muscle following castration, 512
Bicipital synovitis, 570
Canine distemper, experimental vaccination in, 638
Chloral as an anæsthetic in the horse, 308
Chirol, 571
Cocaine anæsthesia of the spinal cord, 725
Demodectic mange in the dog, treatment of, 307
Diabetic urine from complete atrophy of the pancreas, 512
Distemper, treatment of, 792
Etiological agent of vaccine and variola, 576, 788
Etiology of infectious enteritis and bronchopneumonia in calves, 574
Etiology of epizootic ophthalmia in the ox, 385
and treatment of periodic ophthalmia, 433
Experimental vaccination against canine distemper, 572
Gastro-intestinal affection in the dog and cat, 575
Genesic instinct after incomplete castration, persistence of, 307
Hereditary monstrosity, 381
Hypothermia, 724
Indolent and summer wounds, treatment of, 572
Kerophylocele caused by forging, 510
Liver abscess, polynuclear hyperleucocytosis in diagnosing, 724
Mumps in the dog; cynanche parotidæ, 383
Neurectomy in surgical therapeutics, 513
Normal temperature of the mule, 511
Orthonitrophenylpropionic acid for detecting sugar in the urine, 571
Osteoma of the aponeuroses of the thigh, 511
Parturient apoplexy, iodide of potassium in, 432
Periodic ophthalmia, cause and treatment of, 573
etiology and treatment of, 433
Picricated glycerin in treatment of wounds, 570
Pleurisy in the horse with thoracentesis and saline injections, treatment of, 432
Pleurodynia in the horse, 571
Position of the cow in reducing eversion of the uterus, 576
Primary actinomycosis of the bladder, 384
ostitis of the os pedis, 307
Pulmonary antisepsis in strangles infection, 435
Protargol in purulent arthritis, 513
Rarefying osteitis as a cause of fracture, 383
Retina, laceration of the, 433
Seat of abscesses and fistulæ in arthritis of the foot, 576
Sero-diagnosis of tuberculosis, 509
Shoulder lameness, clinical study of the symptoms of, 381
in cattle from subscapular adeno-tuberculosis, 570
Strongylosis in sheep, treatment of, 639
Testing crude and boiled milk, 724
Extracts from foreign journals (French)—
Therapeutic application of light, 434
Toe-crack, treatment of, 792
Transmission of sarcoptes minor from the cat to bovidæ, 574
Tuberculosis, can the ass coutract, 308
sero-diagnosis of, 509
Ulceration of the pituitary mucous membrane simulating glanders, 510
Vermin on chickens, destruction of, 513

FAIR, W. C., V.S., 618
Feeding dogs in health, on the, 137
Felton, H. B., V.M.D., 488, 556
Female dog, concerning inguinal hernia in the, 409
Fever, the salicylates and alkaloids in rheumatic, 306
to the horse, transmission of aphthous, 384
Fistulæ in arthritis of the foot, seat of abscesses and, 576
Fistulous withers, the treatment of, 515
Fleming, George, necrology, 310, 311
resolutions on, 662
Flower Veterinary Library, a ten thousand dollar endowment for the, 449
Foot, seat of abscesses and fistulæ in arthritis of the, 576
-sore horses in our cities, 415
Forage-poisoning, ergotism, 446
Forging, kerophylocele caused by, 510
Formaldehyde, 152
Formalin in glycerin, 429
Formation of cinchona alkaloids, 424
Foster, J. P., V.S., 158, 175, 457, 597, 651
Fourteenth Annual Report of the Nebraska Agricultural Experiment Station, 644
Fowls, roup in, 720
France, law to regulate the practice of veterinary medicine in, 497
Freeman, F. E., 130, 599
French, Cecil, D.V.S., 1, 44, 137, 161, 284, 299, 373, 409, 423, 477, 498, 726
Frothington, L., 801

GAIN, G. H., M.D.C., 445
Gastric and intestinal disorders of the horse, 69
Gastro-intestinal affection in the dog and cat, 575
Gibb, J. M., V.S., 517, 578
Glanders, 154
treatment of, 246
ulceration of the pituitary mucous membrane simulating, 510
Glandules of the dog, the thyroid gland and thyroid, 1
Glennon, James T., 53
Glick, Miss Vilma, 53
Glycerin, formalin in, 429
in the treatment of wounds, precipitated, 570
Grand Rapids Veterinary College, commencement exercises, 263
Graduates of Wisconsin, Society of Veterinary, 535
Gribble, W. H., D.V.S., 811

HAIRLESS dogs, the dentition of two Mexican, 500
Harger, S. J. J., 122, 148, 170, 307, 381, 432, 509, 533, 570, 638
Harrington, E. T. 598
Harvest, the legislative, 387
Health, on the feeding of dogs in, 137
Heart by suture, treatment of wounds of the, 304
Heat stroke, catarrh of the bowels and, 517
Helmer, Jacob, D V S, 205, 446

Hemiplegia laryngis in the horse, 273, 348
Hemorrhagic pododermatitis, 374, 425
Hendren, S. G., V.M.D., 783
Hereditary monstrosity, 381
Hernia in the female dog, concerning inguinal, 409
Hock lameness, neurotomy in cases of, 430
Hoof diseases, 40, 165, 238, 301, 375, 425
Hope, Christopher, necrology, 254
Horse, chloral as an anæsthetic in the, 308
  diphtheria in the, 360
  gastric and intestinal disorders of the, 69
  hemiplegia laryngis in the, 274, 348
  pleurodynia in the, 571
  Show Association of America, National, 650
    personals, Philadelphia, 473
  transmission of aphthous fever to the, 384
  vomition in the, 245
  with thoracentesis and saline injections, treatment of pleurisy in the, 432
Horses in the Boer war, 464
  in our cities, foot-sore, 415
Horse's foot, a few suggestions as to care and treatment of, 295
Hoskins, W. Horace, D.V.S., 132
Hours, spare, 412
Howe, W. E., 394
How we would run a journal, 736
Huidekoper, R. S., 196, 645, 737, 793
  as a professional man, 757
  as a State board examiner, 758
  physician, surgeon, veterinarian, teacher, author, etc., 753
Human actinomycosis, iodide of potassium treatment of, 567
Hypothermia, 724

Ichthargan, 305
Icthoform and its employment in medical therapy, 306
Ide, A. H., 640
Illinois, Dr. Swain's letter, 310
  Veterinary Medical and Surgical Association, 196, 672
Improved veterinary service United States Army, 57, 114, 190, 252
Iodide of potash, effects of mammary injections of, 532
    treatment of parturient paresis by, 341
  potassium in parturient apoplexy, 432
    treatment of human actinomycosis, 567
Iodine, new solution of, 95
Iowa Agricultural College, Veterinary Department, 255
  and Nebraska Veterinary Medical Association, 334
      President's address at the, 368
Indiana legislation, 329
India, tuberculosis among cattle in, 168
Indolent and summer wounds, treatment of, 572
Infection, pulmonary antisepsis in strangles, 435
  through the digestive tract, 717
Infectious enteritis and bronchopneumonia in calves, etiology of, 574
  ulcer of the vulva of cattle, 89
  vulvar disease of cattle, a bacillus from, 92
Inferior cervical artery, aneurism of the, 515
Influenza and distemper in the dog and cat, the use of thermol in, 556
Inguinal hernia in the female dog, concerning, 409
Injection in the dog, experimental rectal, 373
Inoculation in Louisiana, anthrax and preventive, 613
Intelligence and Education, report of committee on, 488
Intestinal disorders of the horse, gastric and, 69
Italy, the V. S. and public health in, 811

Jay, Robert, M.D.V., 40, 165, 238, 301, 375, 425
Jobson, G. B., V.S., 144, 226
Journal banquet, the, 315
  de med. Vet. et de Zootsch., 574
  how we would run a, 736
Just what should have been done, 388

Kansas City Veterinary College, commencement exercises, 265
Kaupp, B. F., D.V.S., 599, 674, 806
Kelly, William H., 122, 742, 759
Kerophylocele caused by forging, 510
Keystone Veterinary Medical Association, 199, 743, 809
Knowles, M. E., State veterinarian, 758
Koch, Prof. Robert, 718

Laceration of the retina, 433
Lameness, 618
  by the subcutaneous injection of essence of turpentine, treatment of, 379
Langdon, W. C., D.V.S., 247
Laparotomy, 477
Laryngis in the horse, hemiplegia (roaring), 274
Law and Williams, reply to Drs., 452
  James, 249, 532, 801
  to regulate the practice of veterinary medicine in France, 497
L'Echo Vet., 381, 383
Leffmann, Dr. Henry, 222
Legislation, 182, 328, 395, 801
  defeat such, 176
Legislative harvest, the, 387
Legislators, among the, 175
Library, a ten thousand dollar endowment for the Flower, 449
Liegerot, C. P., 154
Light, therapeutic application of, 434
Liniment, new turpentine, 720
Lipomata in a parrot, multiple pure, 299
Liquor carbon deterge", 718
Liver abscess, polynuclear hyperleucocytosis in diagnosis, 724
Loblein, E. L., V.S., 295
Local anæsthetic, chloretone as a, 723
Longacre, E. D., V.S., 67
Louisiana, anthrax and preventive inoculation in, 613, 708
Lowe, J. Payne, 666
  Wm. Herbert, 196
Lungs, osteoma in the, 170
Lyford, C. C., B.S., M.D., D.V.S., 691
Lyman, Charles P., 104

Maine Veterinary Medical Association, 130, 599, 674
Mammary injections of iodide of potash, effects of, 532
Mammitis, persistence of microbes in milk of cows which have suffered from, 430
Man, actinomycosis in, 636
Management of brood mares, the, 158
Man and animals, diseases common to, 24
Mange or scabies in the dog, 150
Manitoba, the Veterinary Association of, 197
Marriages, 53, 442, 737
Marshall, C. J., 65, 221, 462
Martenet, Wm. H., D.V.S., 337
Martin, W. J., M.D.C., 80, 167, 240, 304, 378, 427, 502, 563, 636
Massachusetts Veterinary Medical Association, 598

Maternal dystokia, 495
Maxilla, direct compound comminuted fracture with indirect simple fracture of the inferior, 44
McEachran, William, 703
McGill University, Veterinary Department of, 256, 332
McKenzie, J. C., 125
McKillip Veterinary College, 263, 385, 673
Meat and milk, transmission of tuberculosis through, 683
Meat-juice treatment, zomotherapy or, 96
-inspector, examination for, 53, 392, 649
Median neurectomy, 378
Mediastinum of a rhinoceros, sarcoma of the, 142
Medical standard, 306
Metabolism, effects of salicylates upon, 567
Metritis, puerperal septic, 498
Mexican hairless dogs, the dentition of two, 500
Michener, J. Curtis, V.S., 420
E. Mayhew, V.M.D., 341
Michigan State Veterinary Board, 599
Veterinary Medical Association, 269
ways, 244
Milk, testing crude and boiled, 724
Miller, C., 89
Minnesota State Veterinary Medical Association, 125
Moore, J. O., V.S., 584
R. C., 665
Veranus A., 624
Monats. f. prak. Thierheilk., 309
Monstrosity, hereditary, 381
Morphine poisoning, a new antidote for, 563
Mucous membrane simulating glanders, ulceration of the pituitary, 510
Mule, normal temperature of the, 511
Mullaney, T. F., necrology, 738
Multiple pure lipomata in a parrot, 299
Municipal milk inspection, the problem of, 205
Myers Bros., druggists, 168, 241

NATIONAL disgrace, a, 518
Horse Show Association of America, 650
Navicular sheath, open-joint, 584
Nebraska State Veterinary Medical Association, 598, 674
Iowa and, 334
Veterinary Medical Association, President's address at the Iowa and, 368
Necrology—
Burgess, H. M., D.V.S., 591
Clement, Albert W., D.V.S., 178
Fleming, George, C.B., LL D., F.R.C.V.S., 311
Hagyard, Thomas, V.S., 181
Huidekoper, R. S., veterinarian (Alfort), 793
Mullaney, T. F., V.S., 738
Reinhart, Nathan Evans, V.S., 113
Rook, A. M., V.S., 591
Shannon, Frank T., V.M.D., 738
Shaub, Aaron. E., V.S., 591
Stickney, Josiah H., M.R C.V.S., 177
Wardle, Dr., 99
Williams, William, M.R.C.V.S., 180
Zaner, Lloyd, V.M.D., 526
Neurectomy, double tibic peroneal, 298
in surgical therapeutics, 513
median, 378
ulnar, 97
Neurotomy in cases of hock lameness, 430
New Hampshire legislation, 328
Veterinary Medical Association, 202, 338, 534
New York American Veterinary College, 464
University Alumni, Association of the Veterinary Department of the, 337
New York State, attempted reopening registration, 473
Veterinary Medical Society, 598, 667
Niagara and Orleans County Veterinary Medical Society, 336, 534, 601
Notes, 188, 232, 331, 464, 585
Normal temperature of the mule, 511

ŒSOPHAGEAL obstruction, 390
Officer, the veterinarian as a sanitary, 560
Ohio State Veterinary Medical Association, 124
Ontario Veterinary College, 54, 255, 738
commencement exercises, 258
defence of the, 172
New York State Veterinary College, 172, 394, 452, 530
Oöphorectomy, on the recurrence of estruation in the bitch after, 284
Open joint navicular sheath, 584
Operation in the treatment of impervious urachus, 445
Ophthalmia, etiology and treatment of periodic, 433
periodic, cause and treatment of, 573
Osteoma of the aponeuroses of the thigh, 511
Our 1901 meeting, 648
Oxalic acid, manufacture of, 789
Ox, etiology of epizootic ophthalmia in the, 385

PALMER, H. S., B.S., D.V.S., 361
Pancreas, diabetic urine from complete atrophy of the, 512
Parker, John M., D.V.S., 24
Joseph W., 92
Parotidæ, cynanche, 383
Parrot, multiple pure lipomata in a, 299
Parslow, J. W., V.S., 443, 444
Parturient apoplexy, iodide of potassium in, 432
paralysis, the Schmidt treatment for, 545
paresis by iodide of potash, treatment of, 341
Pearson, Leonard, B.S., V.M.D., 108, 122, 196, 319, 449, 757
Pelvic fractures in the horse, abstract from Professor Hunting's remarks on, 744
Pennsylvania State Board of Veterinary Medical Examiners, 131, 599
examinations, 393
legislation of, 801
Veterinary Medical Association, 131, 270, 398, 458, 472, 535, 598
Pennsylvania's law vindicated, 527
Periodic ophthalmia, etiology and treatment of, 433
Peritonitis, perforation of uterine wall and purulent, 498
Persistence of microbes in milk of cows which have suffered from mammitis, 430
the genesic instinct after incomplete castration, 307
Personals, 67, 134, 203, 272, 340, 406, 474, 544, 603, 675, 747
Peters, Austin, 664
Peters, A. T., 598, 674, 801
Pharmaceutical Journal, 305
Phenol in acute articular rheumatism, 564
Philadelphia horse show personals, 473
return of the senior editor of the JOURNAL to, 309
Philippines, rinderpest in the, 300
Picricated glycerin in the treatment of wounds, 570
Pituitary mucous membrane simulating glanders, ulceration of the, 510
Plaskett, Joseph, 599, 674
Pleuritis and pericarditis, traumatic, 731

Pleurodynia in the horse, 571
Pododermatitis, 238. 301, 375, 425
Pododerm, bruises of the, 40
Poisoning, a new antidote for morphine, 563
-forage, 446
Polyarthritis in the cow, acute, 384
Polynuclear hyperleucocytosis in diagnosing liver abscess, 724
Pope, George W., V.S., 129, 473, 601
Lemuel, Jr., M.D.V., 202, 338
Popular Science Monthly, 428
Position of the cow in reducing eversion of the uterus, 576
Post-mortem examination of a cryptorchid bullock, 389
Poultice, starch, 96
Potash, effects of mammary injections of iodide of, 532
Practice of veterinary medicine in France, law to regulate the, 497
Preliminary observations on skin disinfection and wound infection, 624
President's address, 632, 713
at the Iowa and Nebraska Veterinary Medical Association, 368
Preventive inoculation in Louisiana, anthrax and, 613, 708
Primary ostitis of the os pedis, 307
Principles of stock-breeding, the, 226
Problem of municipal milk inspection, the, 205
Production of balsam Peru, 787
Professional education in Spain, 590
Progress of veterinary literature, 564
Promotions of veterinarians in the Bureau of Animal Industry, 587
Protargol in purulent arthritis, 513
Prussian army, equine diseases in the, 788
Puerperal septic metritis, 498
Pulmonary artery, a case of thrombosis, of the, 797
emphysema in a cow, 47

QUININE extraction, 718
Quinn, Miss Mary E., marriage, 53
Quitman, E. L., M.D.C., 152, 797
Quittor, actual cautery in treatment of cartilaginous, 148

RANCK, E. M., 131, 202, 463, 542, 543, 801
Ravenel, Mazyck P., M.D., 15, 222, 307, 381, 432, 509, 570, 638
Recent veterinary literature on surgery, 18
Recognition, a well-merited, 254
Rec. de Med. Vet., 433, 434, 437, 510, 570, 792
Rectal injection in the dog, experimental, 373
Recurrence of estruation in the bitch after oöphorectomy, on the, 284
Red water, 578
Register, veterinary, 438, 605, 677
Relation of the veterinarian to his profession and the public, 778
Remedy for vice, spaying as a, 640
Report of an autopsy, 597
Committee, A.V.M.A., 664
Reports of cases—
Bitch, extra-uterine fœtation in the, 726
Cæsarean section on a cow, 444
Catarrh of the bowels and heat stroke, 517
Cryptorchid bullock, congenital malformations and pathological conditions, post-mortem examination of a, 389
Ergotism, 446
Fistulous withers, the treatment of, 515
Forage-poisoning, 446
Hyposulphite of soda, poisoning, 443
Œsophageal obstruction, 390
Open-joint navicular sheath, 584
Operation in the treatment of impervious urachus, 445
Pulmonary emphysema in a cow, 47
Reports of cases—
Red water, 578
Rupture of diaphragm, 391
Spaying mares as a remedy for vice, 640
Thrombosis of the pulmonary artery, a case of, 797
Traumatic pleuritis and pericarditis, 731
Ulnar neurectomy, 97
Vomiting in the horse, 245
Report of Committee on Intelligence and Education, 488
Repp, John J., V.M.D., 545, 683, 764
Resolutions, A.V.M.A., 659
Pennsylvania State Veterinary Medical Association, 266
Rose Tree's, 796
Retention of stitches in suppurating wounds, 564
Retina, laceration of the, 433
Return of the senior editor of the JOURNAL to Philadelphia, 309
Revue Pharmaceut, 571
Veterinarian, 307, 383, 385
Reynolds, M. H., 18
Rheumatism, phenol in acute articular, 564
Rhinoceros, sarcoma of the mediastinum of a, 137
Rinderpest in the Philippines, 300
(Roaring) hemiplegia laryngis in the horse, 273, 348
Roberts, Dr. G. H., 598
Rosenberger, Randle C., M.D., 415
Royal Commission, a, 788
Rupture of diaphragm, 391

SALICYLATES upon metabolism, effects of, 567
Sallade, J. W., V.S , 528
Salmon, D. E., D. V. S., 196, 664
Sanders, F. R., M. D. C., 390
Sanitary control work, 739
officer, the veterinarian as a, 560
Sarcoma of the mediastinum of a rhinoceros, 142
Sarcoptes minor from the cat to bovidæ, transmission of, 574
Scabies, epicarin in the treatment of, 78
in the dog, mange or, 150
Schmidt treatment for parturient paresis, 545
Schuylkill Valley Veterinary Association, 66
Science of pharmacy, the, 789
Semi-annual meeting of the Veterinary Medical Association of New Jersey, 600
Semimembranous muscle following castration, atrophy of the, 512
Senior editor of the JOURNAL to Philadelphia, return of the, 309
Septic metritis, puerperal, 498
Sero-diagnosis of tuberculosis, 509
Shannon, Frank T., necrology, 738
Shaw, Walter, V.S., 778
Sheath, open-joint navicular, 584
Sherwood, Thomas G., 672
Short-horn breeders toward the tuberculin test, and the animus thereof, the attitude of some, 109
Shoulder lameness in cattle from subscapular adenotuberculosis, 570
Simple fracture of the inferior maxilla, direct compound comminuted fracture with indirect, 44
Skin disinfection and wound infection, preliminary observations on, 624
Sloan, J. A., B.Sc., M.D.V., 40, 165, 238, 301, 375, 423
Soaps, antiseptic, 514
Society notes, 811
Society of Veterinary Graduates of Wisconsin, 535, 598
Society proceedings—
Allegheny County Veterinary Medical Association, 673, 743

Society proceedings—
American Veterinary Medical Association, 745, 810
Association News, 463
Illinois Veterinary and Surgical Association, 196, 672, 807
Iowa and Nebraska Veterinary Medical Association, 334
Keystone Veterinary Medical Association, 199, 462, 543, 743, 809
Maine Veterinary Medical Association, 130, 674
Manitoba, the Veterinary Association of, 197
Maryland State Veterinary Medical Society, 337
Michigan Veterinary Medical Association, 269
Minnesota State Veterinary Medical Association, 125
Missouri Veterinary Medical Association, 674, 805
New Hampshire Veterinary Medical Association, 202, 338
Nebraska State Veterinary Association, 674
Niagara and Orleans County Veterinary Medical Society, 336, 601
New York State Veterinary Medical Society, 667
University Alumni Association of the Veterinary Department of the, 202, 337
Ohio State Veterinary Medical Association, 124, 811
Ontario Veterinary Association, 65
Pennsylvania State Board of Veterinary Medical Examiners, 131, 196
Veterinary Medical Association, 62, 131, 270, 338, 398, 458
Schuylkill Valley Veterinary Association, 66
Tennessee Veterinary Medical Association, 674
U. S. Army Improved Veterinary Service, 57, 114, 190
University of Pennsylvania, Veterinary Department, Society of the Alumni, 539
Veterinary Medical Association of New Jersey, the, 126, 139, 600
Wisconsin Society of Veterinary Graduates of, 537
Wolverine Veterinary Medical Association, 536
Soda-poisoning, hyposulphite of, 443
Solution, Burrow's antiseptic, 95
of iodine, new, 96
Some thoughts on foot-sore horses in our cities, with a view to ameliorate or prevent the same, 415
Spain, professional education in, 590
Spare hours, 412
Spavin, its etiology and treatment, 80
Spaying mares as a remedy for vice, 640
Spinal cord, arteric sclerosis in the, 788
cocaine anæsthesia of the, 725
Sputum, the action of solar and diffuse light upon the tubercle bacillus in, 572
Starch poultice, 96
State Board of Veterinary Medical Examiners, 741
our, 791
Stewart, S., 251, 451, 530
Stitches in suppurating wounds, retention of, 564
Stock-breeding, the principles of, 226
yards, the U. S. inspector in our large, 781
Stomach, transplantation of a piece of colon into the, 429
Strongylosis in sheep, treatment of, 639
Subject, offers himself as a tuberculosis, 577
Subscapular adenotuberculosis, shoulder lameness in cattle from, 570
Sugar in the urine, orthonitrophenylpropionic acid for detecting, 571
Suggestions as to care and treatment of the horse's foot, a few, 395
Summer wounds, treatment of indolent and, 572
Surgery, recent veterinary literature on, 18
Surgical therapeutics, neurectomy in, 513
Surprise, a, the JOURNAL banquet, 315
Suture of arteries, 169
Swain, S. H. V.S., 52, 310, 327
Swain, W. A., 197, 673
Sweetapple, C. H., V.S., 42, 66, 389
Swine, castration tumors in, 233
Sydenham, Thomas, 567
Sympathy wasted, 646
Synovitis, bicipital, 570
Systemic secretion of the testicle, 501

TANNOCREOSOFORM, 304
Tapeworms in a dog, unusual number of, 788
Temperature of the mule, normal, 511
Tennessee Veterinary Medical Association, 599, 675
Ten thousand dollar endowment for the Flower Veterinary Library, 449
Testicle, systemic secretion of the, 501
Testing crude and boiled milk, 724
Text-book of Veterinary Medicine, 566
Therapeutical and pharmaceutical notes—
Absorption of various drugs in the bladder, 719
Actinomycosis in man, 636
Adulteration of cinnamon bark, 718
Alcohol as a disinfectant, 241
Amyl salicylate, 305
Antidote for morphine poisoning, a new, 563
Antiseptics, a comparison of, 720
Burrow's antiseptic solution, 95
Dairy bacteria, 427
Digitalis, 502
Disastrous explosion, 240
Drug adulterations, 94
Effects of salicylates upon metabolism 567
Elimination of antipyrine, 567
Ephemeris, the, 104
Equine diseases in the Prussian army, 788
Etiology of yellow fever, 305
Formalin in glycerin, 429
Hydrogen dioxide for burns, 789
Ichthargan, 305
Ichthoform and its employment in medical therapy, 306
Iodide of potassium treatment of human actinomycosis, 567
Liquor carbon detergen, 718
Manufacture of oxalic acid, 789
Median neurectomy, 378
New solution of iodine, 96
turpentine liniment, 720
Neurotomy in cases of hock lameness, 430
Operations in hairy regions, 380
Oxalic acid, manufacture of, 789
Persistence of microbes in milk of cows which have suffered from mammitis, 430
Phenol in acute articular rheumatism, 564
Pneumococcic arthritis, 243
Preservatives in antitoxin, 242
Production of balsam Peru, 787
Professor Robert Koch, 718
Progress of veterinary literature, 564
Quinine extraction, 718
Resistance of bacteria to heat and cold, 241
Retention of stitches in suppurating wounds, 564
Roup in fowls, 720
Royal commission, 788

Therapeutical and pharmaceutical notes—
Salicylates and alkalies in rheumatic fever, the, 306
Scarcity of sponges, 241
Science of pharmacy, 789
Starch poultice, 96
Sterilizing sponges, 563
Suture of arteries, 169
Sydenham, Thomas, 567
Tannocreosoform, 304
Tapeworm in a dog, unusual number of, 788
Text-book of Veterinary Medicine, 566
Transplantation of a piece of colon into the stomach, 429
Treatment of lameness by the subcutaneous injection of essence of turpentine, 379
Tropacocaine hydrochlorate, 719
Tubercular arthritis, 169
Tuberculosis among cattle in India, 168
Uncertainty surrounding the actions and uses of many of the alkaloids, 167
Unusual number of tapeworms in a dog, 788
Veratrum viride, 428
Villates solution, 787
Zomotherapy, or meat-juice treatment, 96
Therapeutic application of light, 434
Gazette, 244, 304, 638, 723
Therapeutics, neurectomy in surgical, 513
Thermol in influenza and distemper in the dog and cat, the use of, 556
Thierarztliches Centralblatt, 792
Thigh, osteoma of the aponeuroses of the, 511
Thirty-seventh Annual Convention, Detroit, September, 1900, 55
Thomassen, Prof. M. W. J. P., 348
Thrombosis of the pulmonary artery, a case of, 797
Toe-crack, treatment of, 792
Torrance, F., 199
Thyroid gland and thyroid glandules of the dog, 1
Transmission of aphthous fever to the horse, 384
tuberculosis through meat and milk, 683, 762
sarcoptes minor from the cat to bovidæ, 574
Transplantation of a piece of the colon into the stomach, 429
Traumatic pleuritis and pericarditis, 731
Treatment of azoturia, 170
cartilaginous quittor, actual cautery in the, 148
distemper, 792
fistulous withers, 515
human actinomycosis, iodide of potassium, 567
impervious urachus, operation in the, 445
indolent and summer wounds, 572
parturient paresis by iodide of potash, 341
paralysis, the Schmidt, 545
periodic ophthalmia, etiology and, 433
causes and, 573
pleurisy in the horse, with thoracentesis and saline injections, 432
of scabies, epicarin in the, 78
of strongylosis in sheep, 639
of toe-crack, 792
of wounds of the heart by suture, 304
picricated glycerin in the, 570
spavin, its etiology and, 80
zomotherapy or meat-juice, 96
Tropacocaine hydrochlorate, 719
Tubercle bacilli by cows in coughing a possible source of contagion, the dissemination of, 15
bacillus in sputum, the action of solar and diffuse light upon the, 572
Tuberculosis among cattle in India, 168
human and bovine, in the domestic animals, experimental, 33
of man and cattle, 521
subject, offers himself as a, 577
through meat and milk, transmission of, 683, 764
the British Congress on, 586
sero-diagnosis of, 509
Tubercular arthritis, 169
Tuberculin test, cattle inspection and the, 144
the attitude of some short-horn breeders toward the, 109
" R," 242
Tumors, 696
in swine, castration, 233

Ulceration of the pituitary mucous membrane simulating glanders, 510
Ulcer of the vulva of cattle, infectious, 89
Ulnar neurectomy, 97
Underhill, B. M., 412
United States Army, Improved Veterinary Service, 57
Inspector in our large stock-yards, 783
Pharmacopœia, 244
Unusual number of tapeworms in a dog, 788
Urachus, operation in the treatment of impervious, 445
Urine, orthonitrophenylpropionic acid for detecting sugar in the, 571
Uterus, position of the cow in reducing eversion of the, 576

Vaccination against canine distemper, experimental, 572
experimental, 639
Vaccine and variola, etiological agent of distemper, 576, 788
the micro-organism of distemper in the dog, and production of distemper, 802
Van Es, L., M.D., V.S., 273, 348, 759
Variola, etiological agent or vaccine and, 576, 788
Veterinarian as a sanitary officer, the, 560
to his profession and the public, the relation of the, 778
Veterinarians in the Bureau of Animal Industry, 587
needed in the United States army, 650
Veterinary Association of Manitoba, the, 197
Department, Iowa Agricultural College, 255
McGill University, 256
University of Pennsylvania, 533
Society of the Alumni, 539
Journal, 169, 380, 430, 790
library, a ten thousand dollar endowment for the Flower, 449
literature, progress of, 564
Medical Association of New Jersey, 126, 130, 723
medicine in France, law to regulate the practice of, 497
text-book of, 566
organizations and ill-equipped veterinary colleges, 112
move in the matter, let the, 112
Record, the, 804
register, 438, 605, 677, 750, 813
service, United States Army, improved, 57, 114, 190, 252
Surgeon Desmond, 69
Vice, spaying mares as a remedy for, 640
Villate's solution, 787
Vindicated, Pennsylvania's law, 527
Virginia State Board of Veterinary Medical Examiners, 742

Vomition in the horse, 245
Vulva of cattle, infectious ulcer, 89
Vulvar disease of cattle, a bacillus from infectious, 92

Wardle, Dr., necrology, 99
Water, red, 578
Welcome, the Journal's, 524
Wheeler, W. H., V. S., 515
Where in 1902? 794
Williams, Charles, V.M.D., 415
[illegible] William, necrology, 180
Wisconsin Medical Record, 170
  Society of veterinary Graduates of, 537
Wolverine Veterinary Medical Association, 536
Wound infection, preliminary observations on skin disinfection and, 624
Wounds, picricated glycerin in the treatment of, 570
  retention of stitches in suppurating, 564
  treatment of indolent and summer, 572
Wyman, W. E. A., M.D.V., V.S., 40, 78, 97, 165, 238, 245, 301, 375, 425

Year, closing of the, 793

Zeitschrift f. Milchhygiene, 431
Zomotherapy, or meat-juice treatment, 96

